# Reliability of information about the use of antiepileptic drugs during pregnancy from three major web search engines in China

**DOI:** 10.1371/journal.pone.0208783

**Published:** 2018-12-26

**Authors:** Xi Zhu, Xiangmiao Qiu, Dingwang Wu, Shidong Chen, Jiwen Xiong, Hongxuan Du, Zihao Dai, Jamy Hoang, Anjiao Peng, Shixu He, Jianan Duan, Lei Chen

**Affiliations:** Department of Neurology, West China Hospital, Sichuan University, Chengdu, Sichuan, P.R. China; Swansea University, UNITED KINGDOM

## Abstract

**Objectives:**

The objective of this study was to assess the reliability of online information, as provided by three major search engines in China, about the usage of antiepileptic drugs (AEDs) during pregnancy.

**Method:**

Over eight weeks, six physicians conducted a literature search on six computers and six smartphones at a frequency of once per week. During each web search on each computer and smartphone, three major search engines in China were used, namely, Baidu, Sogou and 360. The search terms used were a combination of words, including one AED name (valproate/oxcarbazepine/levetiracetam/lamotrigine) and one Chinese word ("huaiyun" or "renshen", which means pregnancy in Chinese). The top ten websites retrieved from each search were recorded. After the content of each website was evaluated, the sites were categorized into 9 types. Meanwhile, commercial advertisements on each web page were also registered.

**Results:**

A total of 16,411 search results were assessed. After excluding the redundant web pages, 4840 search results were included in the data analysis. Only 12.05% of the search results were reliable, 47.75% were partly reliable, and 40.21% were unreliable. A total of 4139 (85.52%) webpages contained commercial advertisements. The results from a multivariate analysis suggested that websites with no advertisements and professional websites have an independent positive impact on reliability.

**Conclusion:**

Overall, little information on AED usage during pregnancy provided by major search engines in China was reliable.

**Practice implications:**

Accurate and professional online information for female patients with epilepsy should be provided through major efforts by the government, search engine companies, professional websites and epilepsy physicians.

## Introduction

Estimates indicate that at least 65 million people suffer from epilepsy globally [1], and approximately 30% of patients are women of childbearing age [2]. Although the majority of women with epilepsy (WWE) can experience safe pregnancies, both mothers and babies are at an increased risk of poor pregnancy outcomes [3]. Most WWE must continue treatment with antiepileptic drugs (AEDs) and are thus concerned about not only the danger of seizures to both the mother and fetus but also the possible adverse teratogenic events of AEDs, which may result in congenital malformations and neuropsychological impairments. Preconceptual counseling is recommended and necessary for WWE [4], but the situation remains unsatisfactory [5,6], especially in China, where the number of epilepsy specialists is rather inadequate.

WWE are eager to acquire information regarding issues such as the influence of AEDs on their offspring and family planning [6]. Most pregnant women (91.9%) have access to the Internet, and 88.7% of them utilize it as an informational resource to navigate pregnancy-related decisions [7]. With the popularity of computers and smartphones, both devices have become commonly used to search for information. However, patients often have difficulty in judging the quality and accuracy of information and tend to believe and rely on the information they find [8]. Increased demand for reliable medical resources is widespread [9]. It remains unclear whether the most popular web search engine in China could be a source of reliable information about the use of AEDs during pregnancy.

The aim of this study was to investigate the reliability of information about AED usage during pregnancy retrieved by Baidu/Sogou/360, which are the most popular search engines in China after Google's exit from mainland China in 2010 [10]. Given the rapid increase in the number of Chinese mobile Internet users in recent years, we performed the searches on both computers and smartphones. To obtain medium- and long-term search results in a relatively steady manner, we performed the searches once a week for eight consecutive weeks.

## Methods

### Searches

**Fig 1:**
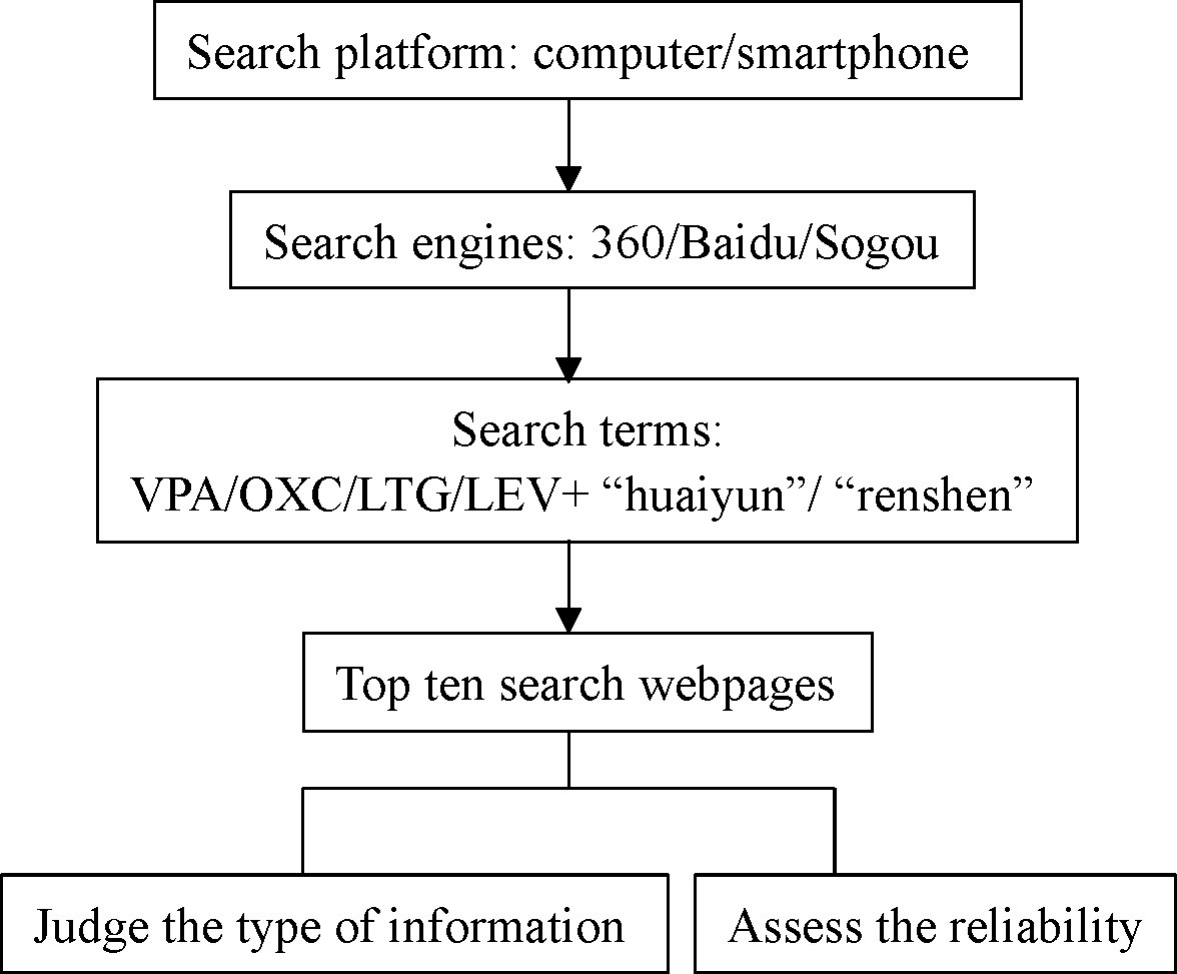
Search flow chart.

Six physicians with expertise in epilepsy from West China Hospital conducted the searches over the course of 8 weeks from November 2016 to January 2017 (for the search process, see Fig 1). Every week, each physician searched the Internet via three different search engines (Baidu, Sogou and 360) on a computer and smartphone simultaneously. The key words for each search were a combination of words in Chinese including an AED name (valproate acid/oxcarbazepine/levetiracetam/lamotrigine) and "huaiyun"/"renshen" (both terms mean pregnancy in Chinese and are commonly used). Therefore, a total of eight pairs of search terms were used. The four types of AEDs were the most widely used AEDs among WWE in China. Moreover, the standard names of the four drugs rather than the branded names were adopted for the search. The top ten websites retrieved in each search were recorded (for a total of 480 web page results, accumulated from each computer and smartphone and the above three search engines by one physician every week). In total, 2880 web pages were browsed every week by six physicians.

Furthermore, all of the engines applied personalized searches, meaning that the presented website ranking is customized and filtered by search history. To avoid this condition as much as possible, we used six different computers and smartphones without signing into search engine accounts and cleared their search histories before conducting each search task.

### Classification of information type

The top ten websites retrieved by each search were opened and evaluated carefully by physicians for classification. Six physicians independently classified the websites according to the contents. The nine categories of websites were online pharmacy websites; hospital websites (homepages of hospitals, mainly private hospitals); web-based encyclopedias (e.g., Baidu encyclopedia, Wikipedia); blogs; instructions for specific drugs; professional websites (academic organizations, academic journals); question-and-answer websites with answers from neurologists (Q&A from neurologists); question-and-answer websites with answers from physicians but not neurologists (Q&A from physicians); and question-and-answer websites with answers from Internet users (Q&A from Internet users). If the web pages could not be opened or the information presented on the websites was irrelevant to use of AEDs among WWE during pregnancy, then the websites were classified as void web pages. Each website was also classified as having or not having advertisements by the physicians. Since a large number of redundant websites could be searched by different physicians, most websites were actually evaluated and classified by more than one physician. Disagreements were resolved by inviting all the six physicians to vote, and the majority vote was used.

### Evaluation of reliability

Reliability was evaluated based on the epilepsy guidelines of the International League Against Epilepsy (ILAE) or the China Association Against Epilepsy (CAAE). If the information provided by the website was totally in accord with the guidelines, the website was evaluated as “reliable”. If not all of the information was correct, the website was rated as “partly reliable”. "Unreliable" referred to websites that were totally incorrect and had apparently erroneous information. Disagreements were resolved by inviting all six physicians to vote, and the majority vote was used. In our study, 91% of the evaluations were consistent.

### Data analyses

Void web pages were not included in the analysis. Variables were reduced to a set of dichotomies to create dummy variables. Thus, a “first ranked variable” was reduced to a variable with a rank of first denoted as 1 and nonfirst rank denoted as 0, a “second ranked variable” had a second rank denoted as 1 and a nonsecond rank denoted as 0, etc. Nominal data were also reduced to dummy variables, including search engines.

Qualitative data are presented as n (%). Univariate and multivariate analysis were used to determine the factors that impacted the reliability of the information provided by websites. Different types of analysis methods were used for different variables in the univariate analysis. Specifically, we used The Wilcoxon rank sum test for ordinal response variables, the Kruskal-Wallis rank sum test for multiple independent rank-order data, and Spearman’s rank correlation analysis for rank-order variables. Furthermore, Bonferroni correction was used to adjust for significant values. Cumulative logistic regression was used to evaluate the independent effect on reliability in the multivariate analysis. Independent variables were screened by the bidirectional stepwise regression method, and the significance level for entering effects was set as 0.30. *P* values of less than 0.05 were considered to be significant. Hypothesis tests were two-sided. Analyses were performed using SAS version 9.4 (SAS institute Inc.).

## Results

A total of 23,040 web pages were retrieved. After excluding 6629 void web pages (defined as web pages that could not be opened or provided irrelevant information), the remaining 16,411 web pages were evaluated and classified by physicians. Among these 16,411 Internet addresses, we identified addresses that occurred more than once and reorganized the information by clustering the observations. A total of 4840 unique sites were identified and included in the data analysis. Among the 4840 sites, 12.05% (583/4840) were determined to be reliable, 47.75% (2311/4840) were partly reliable, and the remaining 40.21% (1946) were unreliable.

Regarding the type of the web pages, online pharmacy websites were the most common type, followed by Q&A from physicians and Q&A from Internet users. Drug instructions, web-based encyclopedias and professional websites were the three least common types (Table 1). Professional websites only accounted for 6.92% (335/4840) of the 4840 web pages. The two search terms “huaiyun” and “renshen” could be found almost equally in most types of web pages, except for professional websites and web-based encyclopedias, in which the term “renshen” was much more common (Table 1). On a total of 505 web pages, the two terms appeared concurrently, resulting in a total of 5345 occurrences of “huaiyun” and “renshen” rather than 4840.

**Table 1:** Sources of online epilepsy information by different search terms.

		Huaiyun [n (%)]	Renshen [n (%)]	Total [n (%)]
Online pharmacy websites		606(12.52)	694(14.34)	1146(23.68)
Q&A from physicians		684(14.13)	546(11.28)	1027(21.22)
Q&A from Internet users		509((10.52)	482(9.96)	890(18.39)
Blogs		254(5.25)	329(6.80)	523(10.81)
Q&A from neurologists		273(5.64)	230(4.75)	410(8.47)
Hospital websites		202(4.17)	283(5.85)	442(9.13)
Professional websites		123(2.54)	240(4.96)	335(6.92)
Web-based encyclopedias		57(1.18)	221(4.57)	253(5.23)
Drug instructions		25(0.52)	35(0.72)	56(1.16)

Univariate analysis was used to determine the reliability of different types of web pages, which showed significant differences in search devices, search engines, search terms, drugs, the presence of advertisements, types of sites, and the ranking of search results (Table 2). After Bonferroni correction and paired comparisons, the results suggested that web pages from Baidu, professional websites, those about Levetiracetam, those ranking in the last 5 among the top ten, those without advertisements, and those using “renshen” were more reliable. A significant difference between computer and smartphone searches was found, with computer searches being more reliable (*P* = 0.0456) in the univariable analysis, while the multivariate analysis showed that the device used had no significant impact on reliability.

**Table 2:** Univariate analysis of reliability in different groups.

		Reliability [n (%)]				*P* value
		Reliable	Partly reliable	Unreliable	Total	
Search devices	Computer	334(14.36)	979(42.09)	1013(43.55)	2326(100.00)	0.0456
	Smartphone	271(10.21)	1392(52.45)	991(37.34)	2654(100.00)	
Search engines	Baidu	474(18.22)	1260(48.44)	867(33.33)	2601(100.00)	<0.0001
	Sogou	84(6.34)	716(54.08)	524(39.58)	1324(100.00)	
	360	39(3.60)	416(38.38)	629(58.03)	1084(100.00)	
Search term	Huaiyun	200(7.99)	1272(50.80)	1032(41.21)	2504(100.00)	0.0006
	Renshen	429(15.1)	1265(44.53)	1147(40.37)	2841(100.00)	
With or without advertisements	With advertisements	285(6.89)	1920(46.39)	1934(46.73)	4139(100.00)	<0.0001
	Without advertisements	298(42.51)	391(55.78)	12(1.71)	701(100.00)	
Drug	Levetiracetam	206(15.57)	658(49.74)	459(34.69)	1323(100.00)	<0.0001
	Oxcarbamacipine	63(5.01)	562(44.67)	633(50.32)	1258(100.00)	
	Valproate	205(17.05)	490(40.77)	507(42.18)	1202(100.00)	
	Lamotrigine	112(9.85)	597(52.51)	428(37.64)	1137(100.00)	
Source of information	Online pharmacy websites	4(0.35)	301(26.27)	841(73.39)	1146(100.00)	<0.0001
	Q&A from physicians	11(1.07)	661(64.36)	355(34.57)	1027(100.00)	
	Q&A from Internet users	8(0.90)	619(69.55)	263(29.55)	890(100.00)	
	Blogs	197(37.67)	304(58.13)	22(4.21)	523(100.00)	
	Hospital websites	4(0.90)	438(99.10)	0(0.00)	442(100.00)	
	Q&A from neurologists	12(2.93)	296(72.20)	102(24.88)	410(100.00)	
	Professional websites	312(93.13)	22(6.57)	1(0.30)	335(100.00)	
	Web-based encyclopedias	59(23.32)	191(75.49)	3(1.19)	253(100.00)	
	Drug instructions	6(10.71)	49(87.50)	1(1.79)	56(100.00)	
Search result rank	Rank 1	23(3.26)	118(16.74)	564(80.00)	705(100.00)	<0.0001
	Rank 2	54(6.72)	243(30.22)	507(63.06)	804(100.00)	
	Rank 3	78(9.77)	317(39.72)	403(50.50)	798(100.00)	
	Rank 4	94(12.84)	362(49.45)	276(37.70)	732(100.00)	
	Rank 5	103(15.01)	371(54.08)	212(30.90)	686(100.00)	
	Rank 6	99(14.89)	366(55.04)	200(30.08)	665(100.00)	
	Rank 7	91(13.13)	442(63.78)	160(23.09)	693(100.00)	
	Rank 8	87(12.48)	449(64.42)	161(23.10)	697(100.00)	
	Rank 9	102(14.19)	458(63.70)	159(22.11)	719(100.00)	
	Rank 10	105(13.80)	497(65.31)	159(20.89)	761(100.00)	

Paired comparison between sources of information showed no statistically significant differences between Q&A from physicians and Q&A from Internet users as well as Q&A from neurologists and Q&A from Internet users. The other paired comparisons were significantly different. The most reliable website type was professional websites, followed by blogs, web-based encyclopedias, drug instructions, hospital websites, Q&A from neurologists, Q&A from Internet users, Q&A from physicians and online pharmacy websites. Significant differences in reliability were found between Q&A from neurologists and Q&A from physicians (*P* = 0.003).

**Fig 2:**
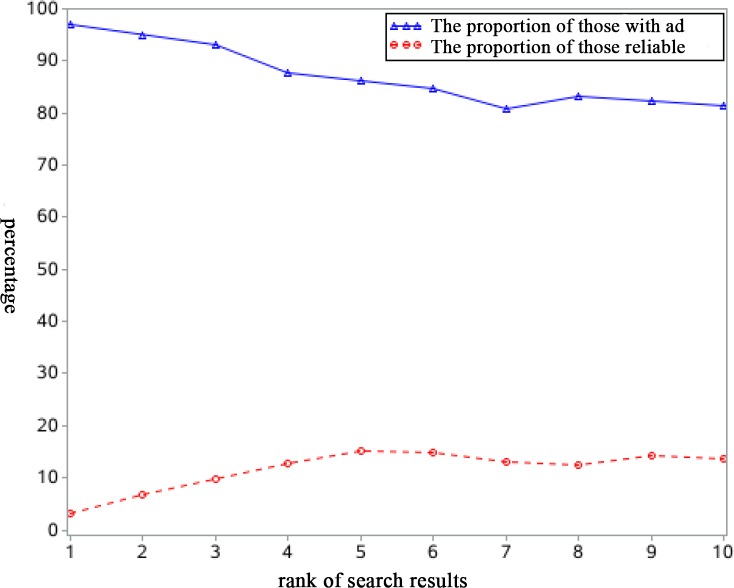
A line graph illustrates that the proportion of reliable websites decreased and commercial advertisements increased as the search result ranks decreased.

Most websites [85.52% (4139/4840)] in the top ten results had advertisements. Interestingly, as search result ranks decreased, the proportion of advertisements decreased, and the web pages seemed to be more reliable (Fig 2).

Multivariate analysis was used to determine the independent factors of reliability and suggested that professional websites had the largest positive impact on reliability (standardized coefficient = 0.95), while hospital websites most negatively impacted the reliability with a standardized coefficient of -0.8447 (Table 3). The reliability of Baidu was compared with that of the other two engines [OR = 1.373, 95% CI (1.122–1.546)]. Websites with no advertisements exhibited a protective effect on reliability [OR = 11.061, 95% CI (8.106–15.093)]. Surprisingly, Q&A from neurologists showed a negative independent effect on reliability with an OR = 0.749 [95% *CI* (0.576–0.972)].

**Table 3:** Independent effect on reliability by cumulative regression analysis.

Independent variables	Coefficient	*P* value	*OR* (95% CI)
Baidu	0.2756	0.0007	1.317(1.122~1.546)
360	-0.2782	0.0042	0.757(0.626~0.916)
With no advertisements	2.4034	<0.0001	11.061(8.106~15.093)
LEV	0.26	0.0016	1.297(1.104~1.524)
Online pharmacy websites	-1.3954	<0.0001	0.248(0.204~0.301)
Hospital websites	-5.3181	<0.0001	0.005(0.002~0.013)
Web-based encyclopedia	1.3063	<0.0001	3.692(2.434~5.600)
Blog	3.3809	<0.0001	29.398(20.749~41.652)
Drug instructions	1.3146	0.0004	3.723(1.798~7.708)
Professional websites	6.8102	<0.0001	907.069(529.549~>999.999)
Q&A from neurologists	-0.2896	0.0299	0.749(0.576~0.972)
Rank 1	-0.9484	<0.0001	0.387(0.302~0.496)
Rank 2	-0.2106	0.0434	0.810(0.660~0.994)

## Discussion and conclusion

### Discussion

Pregnant women increasingly use the Internet as an important source of health information [11]. However, many pregnant women are confused by inaccurate information on websites and, as a consequence, might experience anxiety [12]. To accurately assess the reliability and type of information about on the use of AEDs during pregnancy among WWE via the most popular search engines in China, we gathered and assessed 4840 unique websites.

There are several main findings. First, in most cases, the top ten search results on the main search engines in China were unreliable. Second, professional websites were seldom found among the top ten search results. Third, as the ranks of search results decreased, commercial advertisements increased, and the information became less reliable.

The Internet, with its all-pervasive influence, has been recognized as a potential means for transforming health care. As a well-known way of retrieving health-related information, web search engines enhance information availability and facilitate doctor-patient interaction [13], particularly in some lesser-known subspecialties. However, although the reliability of information and its impact on individuals have remained unclear until recently, the amount of health information on the Internet has been growing on a larger scale, and most users check only the top websites listed on the first page of search results [14]. In such circumstances, the reliability of information is clearly more important than the availability of information. Our study found that much information on websites is useless and even misleading. We found that only 12.05% of the search results were reliable. A similar study found that 19.5% of epilepsy-related information found on the main search engines in China in 2014 was correct and complete [15]. The situation seems dismal in China.

Among the 9 categories, professional websites seem to be most reliable, yet they represent a very small proportion and their contents are often too technical for patients to understand. There are many professional websites, including the China Association Against Epilepsy (http://www.caae.org.cn) and Dingxiangyuan (http://www.dxy.cn), but these professional websites are seldom found in the top ten search results. A similar study found that the representation of the Epilepsy Foundation website was 10% in Google search results [16]. Its representation is clearly higher than that in our study. Q&A websites, which are online counseling services, are currently very popular and seem to have a bright future. However, the reliability of the information provided by Q&A websites is unsatisfactory (e.g., stating that acupuncture and traditional Chinese medicine are better treatments than AEDs). Surprisingly, Q&A websites with answers from physician/neurologists did not offer high-quality information. Physicians lacking knowledge of epilepsy often provided incorrect information, while neurologists who are not familiar with AED usage during pregnancy always provided incomplete information. Moreover, it is worth noting that hospital websites had the greatest negative impact on reliability. Nearly all of these websites were for private hospitals. Interestingly, most private hospitals were hospitals from Putian, a county in Fujian province in the South of China. Approximately 80% of the private hospitals (at least 8000 hospitals) in China are owned by people from Putian [17]. Unlike private hospitals in the West, Chinese private hospitals generally only seek profit and ignore patients' health (e.g., even suggesting that WWE should implant subcutaneous magnets to cure epilepsy). This finding could explain the unreliable information provided by hospital websites.

A large number of the web pages retrieved contained commercial advertisements. Most of the advertisements came from private hospitals and turned out to be specific links to their own websites. These advertisements were full of exaggeration and false claims to lure epilepsy patients into treatment. Investors of private hospitals seek a high financial return and make their websites more accessible by competitively bidding to obtain higher search rankings. Therefore, it is easy to understand why the search results contained so much misleading and inadequate information. The higher the rank of the search results is, the more commercial advertisements they contain and the less reliable they are (Fig 2). In this case, the most typical example is the death of Wei Zexi. Wei Zexi, a university student who died in April 2016 from a rare form of cancer, had sought treatment from a private hospital that appeared at the top of the list on his Baidu web search [18]. However, the treatment he received with an abandoned technique only accelerated his death. The incident caused widespread concern all over the world.

Proper search strategies can increase the probability of obtaining accurate information. Our study showed that the word “huaiyun” was more useful than the word “renshen” for obtaining more credible information. This variability in the results obtained by using different search options highlights the importance of guiding patients to patient-friendly, high-quality websites [19]. Therefore, epilepsy doctors should help patients improve their search strategies to find accurate information.

More than 660 million people use the Baidu mobile search engine every month, meaning that smartphones are an important Internet search tool for WWE. In our study, there was no significant difference between computers and smartphones in the reliability of their content. Previous studies have evaluated differences in the search results of different Google accounts and found some variability in the top 3 results, but the overall differences were different than expected [16]. In this study, we did not use the sign-in accounts of the engines in order to avoid the influence of the sign-in web history. Although Baidu is the largest search engine company in China and is frequently enmeshed in scandals in the medical field [20], it was still more reliable than the other two search engines.

### Limitations

Our study has a limitation in that we used three main search engines to examine the reliability of website information yet were unable to include Google, the search engine used throughout the world. Health care information from Google might result in a different conclusion, and AED-related information might be more reliable.

### Conclusion

In conclusion, as an important part of e-health, web search engines are a very efficient and low-cost way of obtaining health care information [21]. However, most current information on AEDs used by pregnant women is not only unreliable but also limited by the major search engines in China. Some information, largely provided by private hospitals, may mislead patients to obtain inappropriate treatments due to the perceived economic benefits.

### Practice implications

Accurate and high-quality web information should be provided for WWE though joint efforts by the government, search engine companies, professional websites and epilepsy specialists [22]. Professional websites should provide more high-quality information and implement search engine optimization to obtain better rankings. Governmental regulations are also needed to prevent competitive bidding from becoming excessive in the medical field and guiding patients in the wrong direction. Training on WWE should be improved among physicians and neurologists, as well as among epilepsy specialists.

## Supporting information

S1 File Data underlying the findings described in our study. (XLSX)
